# Interference mitigation in intentional jammers aided non-uniform heterogeneous cellular networks

**DOI:** 10.1371/journal.pone.0287709

**Published:** 2023-06-28

**Authors:** Saleh Mohammed Ghonaim, Samar Khan, Faisal Althobiani, Shadi Alghaffari, Sheraz Khan, Muhammad Irfan, Muhammad Sajid Haroon, Fazal Muhammad

**Affiliations:** 1 Nautical Science Department, Faculty of Maritime, King Abdulaziz University, Jeddah, Saudi Arabia; 2 Department of Electrical Engineering, University of Engineering and Technology, Mardan, Pakistan; 3 Marine Engineering Department, Faculty of Maritime, King Abdulaziz University, Jeddah, Saudi Arabia; 4 Port and Marine Transportation Department, Faculty of Maritime, King Abdulaziz University, Jeddah, Saudi Arabia; 5 Electrical Engineering Department, College of Engineering, Najran University, Najran, Saudi Arabia; 6 Telecommunications and Networking (TeleCoN) Research Lab, GIK Institute of Engineering Sciences and Technology, Topi, Pakistan; Thamar University: Dhamar University, YEMEN

## Abstract

Coverage and capacity are optimized in fifth generation (5G) networks by small base station (SBS) distribution in the coverage realm of macro base station (MBS). However, system performance is significantly reduced by inter-cell interference (ICI) because of the orthogonal frequency division multiple access assumption. In addition to ICI, this work considers intentional jammers’ interference (IJI) due to the presence of jammers. These Jammers try to inject undesirable energies into the legitimate communication band, which significantly degrade uplink (UL) signal-to-interference ratio (SIR). To reduce ICI and IJI, in this work, we employ SBS muting, where the SBSs near MBS are switched off. To further mitigate ICI and IJI, we use one of the effective interference management schemes a.k.a reverse frequency allocation (RFA). We presume that due to mitigation in ICI and IJI, the UL coverage performance of the proposed network model can be further improved.

## 1 Introduction

### 1.1 Motivation

Heterogeneous cellular networks (HetNets) is a promising candidate technology for the future fifth generation (5G) networks [1–3]. The world wireless research forum predicts high speed connectivity for trillion of devices in the near future [4]. 5G networks can achieve capacity of 100 Gbps with improved battery life, higher coverage, and enhanced user accommodation [5, 6]. HetNets are made up of tiny, small base stations (SBS) coupled with high-power macro base stations (MBS) from a homogeneous cellular network [2, 7]. The deployment of such base stations (BSs) enhances network scalability [8, 9].

Intra-cell interference(ICI) is still the key limiting factor in HetNets despite the adoption of orthogonal frequency division multiple access (OFDMA), which results in minimal intra-cell interference [10, 11].

The functioning of the HetNet network can be negatively impacted by severe intentional jammers’ interference (IJI) caused by jammers’ presence [12–14]. The location of base stations transmit power, and other network parameters are all presumptively known to such jammers [13]. Therefore, by introducing undesired energy in the appropriate communication range, they can significantly degrade the uplink (UL) signal-to-interference ratio (SIR) [13]. Due to (i) decreased MBS-edge user (M-EU) power transmission in UL, (ii) greater M-EU user distances, and (iii) a higher path-loss exponent, IJI is effective in UL [14, 15].

SBS muting is taken into consideration in HetNets because I a user receives more coverage close to the MBS [5] and (ii) a higher MBS transmit power causes significant co-tier interference [16]. Due to less SBS deployment, SBS muting results in lower ICI and IJI, which enhances network coverage [17].

We refer to SBS muting by non-uniform HetNets (NUHs) and without SBS muting by uniform HetNets (UHs) in the remaining sections of the work. Different interference mitigation strategies, including reverse frequency allocation (RFA) [18], cell range extension (CRE)(CRE) [19], and fractional frequency reuse (FFR) [20], are used in the state-of-the-art. RFA is regarded as one of these plans’ proactive and effective interference mitigation strategies [18, 21].

Different key 5G technologies with their applications are presented in [22, 23]. Latest work on HetNets along with emerging technologies, such as (i) non-orthogonal multiple access (NOMA), (ii) massive multiple input multiple outputs (massive MIMO), and (iii) millimeter wave can be found in [24–26].

The works in [27, 28] evaluate both intra-cell interference and ICI in 5G networks. The authors used inter-cell interference coordination (ICIC) technique to mitigate the interference. Through results, it is shown that ICIC leads to improved network performance results. Similarly, in [29], the authors investigate the inter-block interference (IBI) and ICI in HetNets. They propose a novel precoding scheme to reduce ICI and IJI in HetNets.

Their proposed model leads to significant performance superiority due to lower IJI and ICI. The above-mentioned work, however, lacks to investigate both SBS muting and RFA scheme.

The work in [30] study the security aspects of 5G networks focusing on various types of attacks and security services. Moreover, security concerns are evaluated for different 5G technologies, such as software-defined networks, device-to-device communications, heterogeneous networks, massive MIMO, and the Internet of things. Moreover, attacks on 5G networks including traffic analysis, eavesdropping, denial of service, distributed denial of service, and jamming are investigated. The study in [31], provides an in-depth analysis of different jamming and anti-jamming techniques in 5G networks. Similarly, the works in [32] investigate the spoofing and jamming of the physical downlink and UL control channels and signals in 5G networks. Moreover, they employ various jamming methods to evaluate network immunity against jamming. They conclude that effective measures are needed to mitigate jamming in 5G networks. In contrast to our work, [30–32] lacks the employment of RFA and SBS muting to reduce ICI and IJI.

In [33], the authors employ NOMA enabled NUH, where SBSs are distributed with different densities in various regions. Through results, they demonstrate that NOMA-enabled NUH outperforms all other scenarios in terms of energy efficiency. Similarly, the works in [34, 35] explore the employment of NUHs. Their results indicate significant performance improvement due to lower interference achieved by SBS muting in HetNets. However, the latest work of [33–35] lacks to analyze IJI in HetNets.

The latest work on RFA employment can be found in [17, 21, 36], where RFA scheme leads to better coverage and rate due to effective mitigation of interference. However, they lack to investigate both NUH and intentional jammers in HetNets.

In this work, we look into HetNets’ performance in terms of coverage when there is IJI and ICI. To alleviate the impact of ICI and IJI we use SBS muting as well as RFA as a preventive measure for interference mitigation.

### 1.2 Approach and contributions

**Fig 1:**
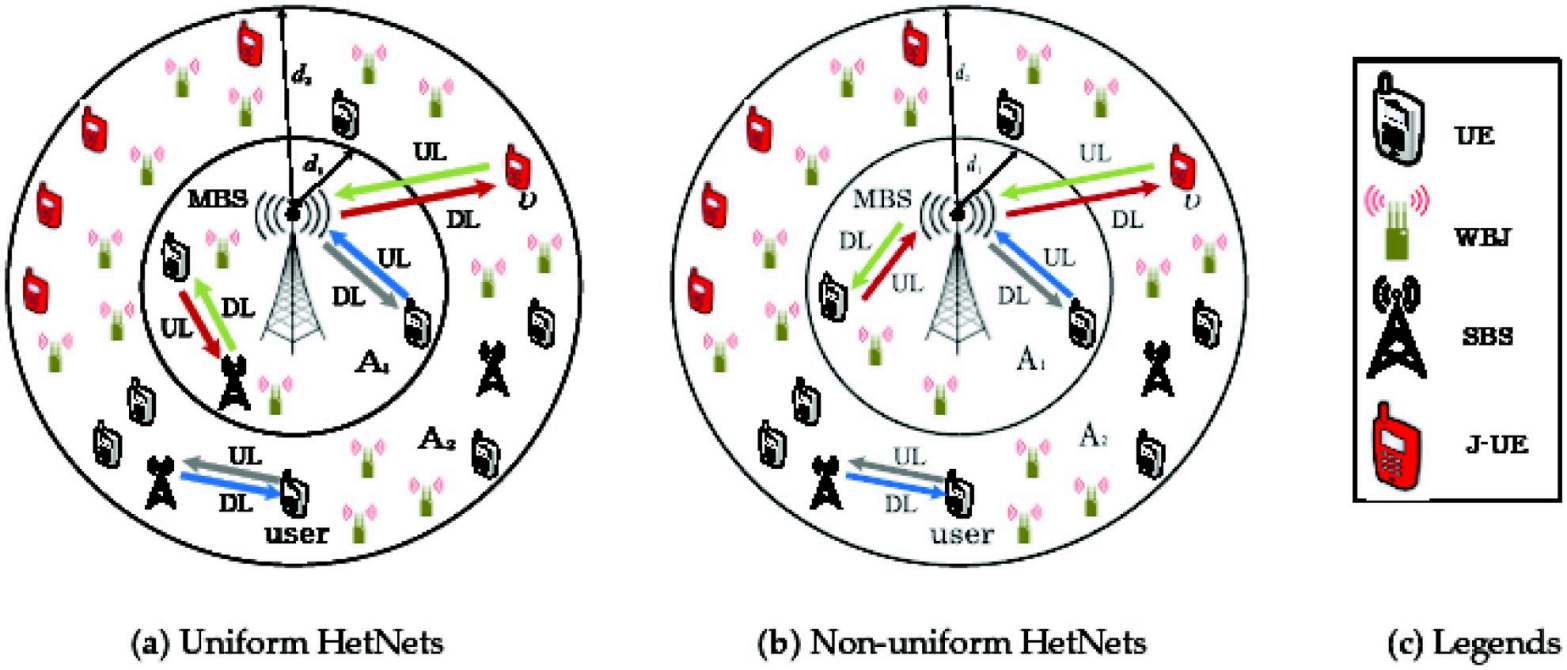
The proposed framework of HetNet that incorporates. A: Uniform HetNets. B: Non-uniform HetNets. C: Legends.

The paper uses two layers of BS, known as MBSs and SBSs, to illustrate a model based on HetNets. IJI attacks frequently result in additional UL intersections in addition to the typical ICI. Thus, the system as a whole is considered degenerative. In Fig 1A and 1B. the network models are displayed. The MBS service area is divided into two sections: the inner zone, designated *A*_1_, and the edge region, designated *A*_2_, with radii Δ*s*_1_ and Δ*s*_2_, accordingly [37, 38].

The significance of this work from the state-of-the-art can be summarized as follow.

1. The work in [27–29] evaluates both intra-cell interference and ICI in 5G networks. However, they lack to investigate IJI.
2. In contrast to our work, [30–32] lacks the employment of RFA and SBS muting to reduce ICI and IJI.
3. The latest work of [33–35] evaluates NUHs but lacks to analyse IJI in HetNets.
4. The latest work on RFA employment can be found in [17, 21, 36]. However, they lack to investigate both NUH and IJI in HetNets.

The following are this paper’s significant contributions.

1. Analysis of the UL coverage for the typical user which is defined as Slivnyak theorem states that the statistical characteristics of an independent homogeneous Poission Point Process (IHPPP) are preserved and simplified by a typical user at origin [39, 40]., U, in *A*_2_ when IJI and ICI are present.
2. This study examines how proactive interference control strategies can reduce IJI and ICI. RFA and the use of NUHs, a smart network design technique.
3. For (i) UHs with RFA employment (see (10)) and (ii) NUHs with RFA employment (see (11)), we develop coverage probability expressions.
4. The outcomes are presented for various network characteristics, including SIR threshold, jammers’ density and transmitted power, users’ transmit power and density of SBS.

### 1.3 Paper organization

The remainder of the paper is laid out as follows. The system model is presented in Section 2. The suggested model’s coverage probabilities are calculated in Section 3. Section 4 contains the results and commentary. The paper is finished in Section 5. Table 1 showcases a qualitative tabulation of various references that carried work based on our proposed work that needs improvements while, Table 2 contains an index of the notations made in the article. And finally, Table 3 has the system parameters defined.

**Table 1:** Qualitative reference table.

S.No.	Reference	Methodology	Technique	Benefits	Drawbacks
1	[12]	Heterogeneous Wireless network model (HWNs)with nodes of each tier are located and deployed in PPP with intensities known.	Expressions are derived for random multitier HWNs where joint blackhole jamming attacks exist.	Detection and avoiding association of end users with malicious nodes such as jammers and blackholes.	5G is susceptible to jamming attacks leading to legitimate user coverage interference.
2	[13]	Key parameters of 5G are discussed especially various channels and exchanges between signals over equipment and base stations.	Jamming attack detection such as packet delivery and drop ratios etc., while using the threshold of the defined metrics.	Jamming attack mitigation through frequency hopping spread spectrum(FHSS) and direct sequence spread spectrum (DSSS).	DSSS is capable of achieving protection of high degrees.
3	[14]	Adaptive bias configuration strategy is presented for range extension (RE) through cell load balancing.	Dynamic adaptive bias value is set in accordance to the environmental changes.	RE has the potential to avail low-powered node resource efficiently as well as effectively through cell edge performance.	If bias value is not set properly, interference may increase.
4	[15]	Decoupling association (DeCA) is used for MBS M-EUs to improve UL SIR.	DeCa with RFA	Wide-band jammer (WBJ) cluster severely reduces the UL communication.	Jammer density and transmit power degrades network.
5	[16]	Ground-to-air offloading and BS coordination scheme to enhance mobile users (MUs) performance.	Network throughput, Average spectral efficiency (SE), and analysis through a theoretical framework.	Simulations and numerical analysis validate the impact of key system parameters on system performance demonstrating UAV-assisted offloading scheme advantages.	Flying UAVs require a power source.
Our Work	[39]	RFA in NUH with non-uniform BSs deployment.	RFA	ICI and IJI mitigation to enhance UL performance of M-EU’s.	Wastage of SBS resources and system performance degrades due to OFDMA.

**Table 2:** Notation summary.

Notation	Description
*ϕ*_M_, *ϕ*_S_, *ϕ*_j_	IHPPPs of MBSs, SBSs, and jammers, respectively
*ν*	Typical user
Γ_M_	SIR threshold for MBS
Δ*s*_1_, Δ*s*_2_	Radii of *A*_1_ and *A*_2_, respectively
$P_{t,\nu }^{\text{UL}}$	UL transmit power of *ν*
*ζ*_M_, *ζ*_S_, *ζ*_j_	Densities of uniformly distributed MBSs, SBSs and jammers, respectively
*α*	Path loss exponent, ∀ *α*_M_ = *α*_S_ = *α* and *α* > 2
|*h*_*l*_|, |*h*_*k*_|, |*h*_*j*_|	Power gain Rayleigh fading of MBS, SBS and jammers, respectively
*r*_*l*_, *r*_*k*_, *r*_*j*_	distances from MBSs, SBSs, and jammers, respectively ∀ *l* ∈ {*ϕ*_M_}, *k* ∈ {*ϕ*_S_}, and *j* ∈ {*ϕ*_*j*_}
${\text{SIR}}_{\text{M}}^{\text{UL}}$	Uplink SIR received by MBS
UL, DL	Uplink and Downlink, respectively
L	Laplace transform parameter
*η*_1_	Ratio of $P_{\text{t,S}}^{\text{DL}}\;\text{and}\;P_{t,\nu }^{\text{UL}}$
*η*_2_	Ratio of $P_{t,j}\;\text{and}\;P_{t,\nu }^{\text{UL}}$

**Table 3:** Simulation parameters.

Parameter	Configuration
MBS, SBS, and IJs	IHPPP
Channel bandwidth	10 MHz
No. of iterations in simulation	10000
Δ*s*_1_, Δ*s*_2_	600 and 1000 m, respectively
*ζ*_S_	90 / *π*(500m)^2^ [35]
*ζ*_M_	3 / *π*(500m)^2^ [39]
*ζ*_*j*_	15 / *π*(500m)^2^ [39]
$P_{t,\text{M}}^{\text{DL}}$ , $P_{t,\text{S}}^{\text{DL}}$,*P*_*t*,*J*_, $P_{t,u}^{\text{UL}}$	40 dBm, 30 dBm, 20 dBm and 20 dBm, respectively [48]
*α*_*m*_ = *α*_*s*_ = *α*	2 < *α* ≤ 4 [49]

## 2 System model

This section presents the suggested network design as shown in Fig 1A and 1B. Due to multi-tier BSs deployment and the existence of intentional jammers the network performance degrades severely due to ICI and IJI. UL communication of M-EUs in HetNets are susceptible to IJI and ICI because of lower UL transmit power and longer transmission distances between MBS and M-EUs. Moreover, we incorporate RFA in NUH with non uniform BSs deployment to mitigate both ICI and IJI and thus, enhance UL performance of M-EUs. Preliminary mathematical results obtained in this section are used for coverage probability assessment in Section 3.

### 2.1 Network layoutS

In this paper, we consider two-tier HetNet comprising of co-deployed SBS’s with MBS’s. We suppose that there exist intentional jammers throughout the network which degrade the desired communication link. MBS’s, SBS’s, users, and jammers are distributed via IHPPPs *ϕ*_M_, *ϕ*_S_, *ϕ*_*u*_, and *ϕ*_*J*_, respectively [17]. The density of MBSs, SBSs, users, and jammers is *ζ*_M_, *ζ*_S_, *ζ*_*u*_, and *ζ*_J_, respectively. The proposed network models are presented in Fig 1A and 1B [17, 18, 33]. We assume that the UL communication of M-EUs are stressed by IJI and ICI. This work assumes NUHs with RFA in contrast to UHs with RFA to reduce ICI and IJI. Moreover, we investigate the UL coverage performance of U located in *A*_2_. The path loss exponent is denoted by *α* [41, 42]. The Rayleigh fading gain, i.e., |*h*|^2^ ∼ exp(1) [17, 40] is represented by |*h*|. For RFA and NUH employment, we divide the MBS coverage region in to *A*_1_ and *A*_2_ with radii Δ*s*_1_ and Δ*s*_2_, respectively [37, 43].

### 2.2 Jamming mechanism

Jammers are considered to transmits unwanted energy across the entire spectrum of the communication system to reduce network performance [37, 38, 44]. This work assumes that the jammers are located uniformly in the coverage vicinity of MBS which are distributed according to IHPPP [35, 45]. The UL communications of M-EUs in HetNet is significantly degraded by ICI and IJI [39]. Due to power constraints, jammers in lower density or located at far distance merely cause any harm to the communication system [15]. Therefore, such low power jammers to be effective, they must be well tuned and need to be located near the target [45, 46]. Moreover, in worst case scenario, jammers block the UL communication in HetNets and, thus, cause the distributed denial of service (DDoS) attacks [39, 45].

### 2.3 Reverse frequency allocation

**Fig 2:**
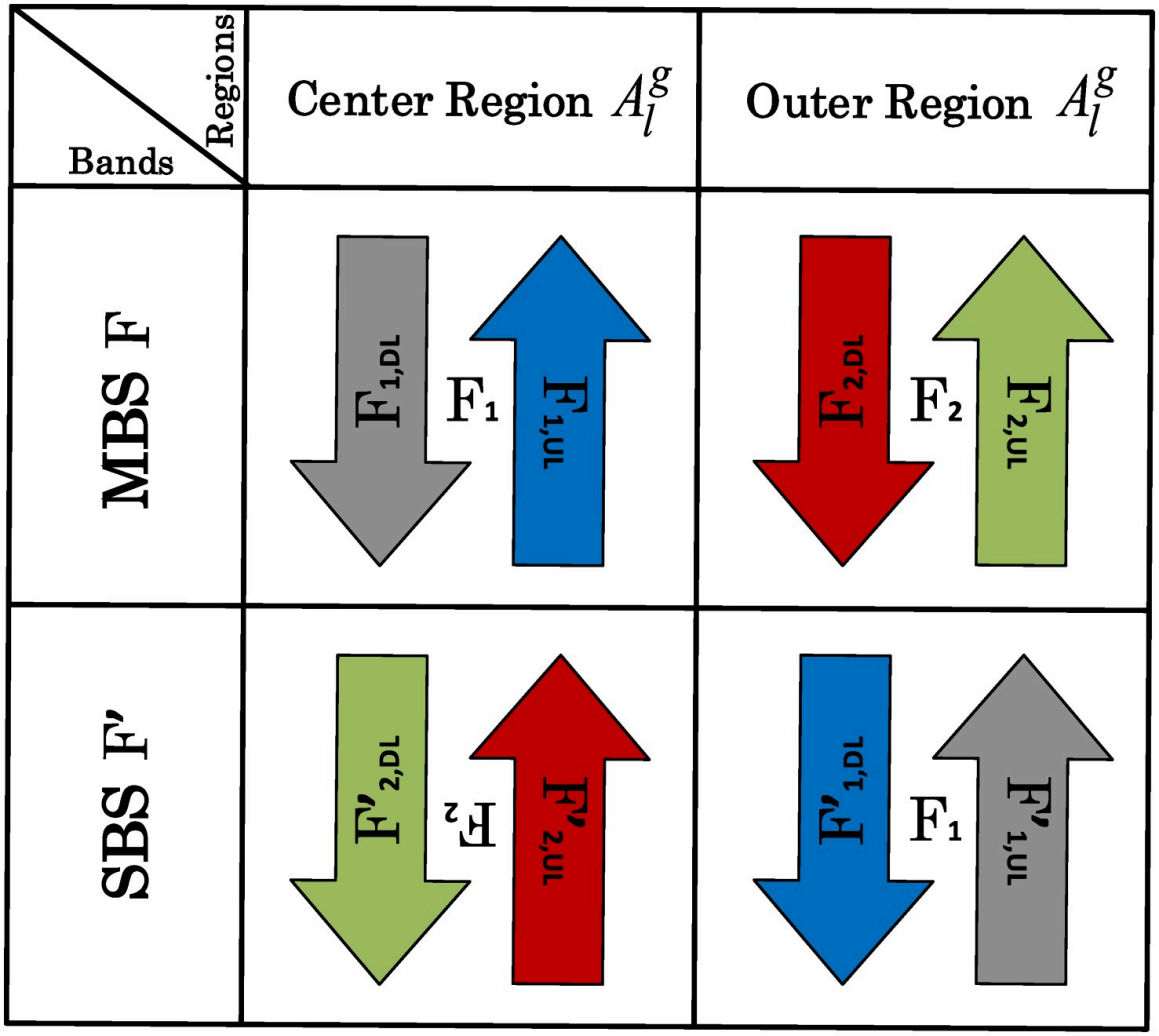
RFA architecture in HetNets.

Due to efficient interference mitigation, RFA-based resource partitioning significantly improves coverage [39]. By using RFA, the entire spectrum is left open for an SBS to use in the opposite direction and in non-overlapping regions [18, 39]. Various sub-bands are used interchangeably among SBSs and MBSs while following RFA as $A_{l}^{g}$ ∀ *g* ∈ (1, 2) and *l* ∈ (M, S) used alternatively. Fig 2 showcases this scenario. M stands for MBS, while S stands for SBS.

In-accordance with RFA, total alloted frequency band, F, is further divided into sub-bands with different frequencies, i.e., F_1_ and F_2_, such that F = ⋃_*z*∈(1,2)_ F_*z*_, as shown in Fig 2. Whereas, these sub-bands F_1_ and F_2_ of MBS is used for UL and DL communication in outer area macro cell ($A_{\text{M}}^{2}$) and inner area of macro cell ($A_{\text{M}}^{1}$), respectively. For the UL and DL communication, these sub-bands are further split into UL and DL sub-carriers which are modeled as F_1_ = F_1,UL_+ F_1,DL_ and F_2_ = F_2,UL_+ F_2,DL_, respectively. Similar to F_1_ and F_2_, as sub-band frequencies of MBS, the sub-bands for SBSs are ${\text{F}}_{1}^{\prime }$ and ${\text{F}}_{2}^{\prime }$, respectively, which are reversely used in the corresponding regions reciprocally, i.e., outer region of SBS, $A_{\text{S}}^{2}$, and center region of SBS, $A_{\text{S}}^{1}$, respectively. These sub-bands of SBSs i.e., ${\text{F}}_{1}^{\prime }$ and ${\text{F}}_{2}^{\prime }$, are further cut-up into sub-carriers of UL and DL denoted respectively as ${\text{F}}_{2}^{\prime }={\text{F}}_{2,\text{UL}}^{\prime }+{\text{F}}_{2,\text{DL}}^{\prime }$ and ${\text{F}}_{1}^{\prime }={\text{F}}_{1,\text{UL}}^{\prime }+{\text{F}}_{1,\text{DL}}^{\prime }$ for notation clarity.

## 3 Coverage probability

This section focus on the assessment of coverage probability in the proposed network scenarios where *ν* is assumed to be located in *A*_2_ and in *A*_1_; (i) uplink coverage probability for uniform HetNets (UHs’) is presented in Subsection 3.1 while (ii) the uplink coverage probability in case of non-uniform HetNets (NUHs’) is derived in Subsection 3.2.

### 3.1 Uplink coverage probability for uniform HetNets (UHs)

The UL coverage probability when there are intentional jammers (IJs) and RFA, $P_{A_{2}}^{\text{UL},*}(\Gamma_{\text{M}})$, while considering *ν* in *A*_2_ can be obtained as:

$$\begin{array}{c} \begin{array}{c} P_{A_{2}}^{\text{UL},*}(\Gamma_{\text{M}})=P({\text{SIR}}_{\text{M}}^{\text{UL}}>\Gamma_{\text{M}}). \end{array} \end{array}$$ (1)

Following the architecture of RFA, the total interference in UL is the addition of the UL interference from MBSs in *A*_2_, i.e., $I_{\phi_{\text{M}},A_{2}}^{\text{UL}}$, the DL interference from SBSs in *A*_1_, i.e., $I_{\phi_{s},A_{1}}^{\text{DL}}$, and the interference from IJs, i.e., *I*_*J*,*A*_. Therefore, ${\text{SIR}}_{\text{M}}^{\text{UL}}$ from (1) can be written as:

$$\begin{array}{cc} & {\text{SIR}}_{\text{M}}^{\text{UL}}=\frac{P_{t,\nu }^{\text{UL}}{\left|h_{\text{M}}\right|}^{2}r_{\text{M}}^{-\alpha }}{I_{\phi_{\text{M}},A_{2}}^{\text{UL}}+I_{\phi_{\text{S}},A_{1}}^{\text{DL}}+I_{\phi_{J},A}}. \end{array}$$ (2)

Eq (2) can be expanded as:

$$\begin{array}{c} {\text{SIR}}_{\text{M}}^{\text{UL}}=\frac{P_{t,\nu }^{\text{UL}}{\left|h_{\text{M}}\right|}^{2}r_{\text{M}}^{-\alpha }}{\sum_{l\in \phi_{\text{M}}}\;P_{t,l}^{\text{UL}}|h_{l}{|}^{2}r_{l}^{-\alpha }\;+\;\sum_{k\in \phi_{\text{S}}}\;P_{t,k}^{\text{DL}}|h_{k}{|}^{2}r_{k}^{-\alpha }\;+\;\sum_{j\in \phi_{J}}\;P_{t,j}{\left|h_{j}\right|}^{2}r_{j}^{-\alpha }}. \end{array}$$ (3)

In (3), $P_{t,l}^{\text{UL}}$ is the *ν* UL transmission power connected with MBS, $P_{t,k}^{\text{DL}}$ is the transmission power of SBS, and *P*_*t*,*j*_ is the emitting power of jammers. Moreover, substituting (2) into (1), we obtain $P_{A_{2}}^{\text{UL},*}(\Gamma_{\text{M}})$ as:

$$\begin{array}{c} \;P_{A_{2}}^{\text{UL},*}(\Gamma_{\text{M}})\overset{\left(1\right)}{=}P(\frac{P_{t,\nu }^{\text{UL}}{\left|h_{\text{M}}\right|}^{2}r_{\text{M}}^{-\alpha }}{I_{\phi_{\text{M}},A_{2}}^{\text{UL}}+I_{\phi_{\text{S}},A_{1}}^{\text{DL}}+I_{\phi_{J},A}}>\Gamma_{\text{M}})\\ \overset{\left(2\right)}{=}{\text{E}}_{r_{\text{M}},I_{\phi_{\text{M}},A_{2}}^{\text{UL}},I_{\phi_{\text{S}},A_{1}}^{\text{DL}},I_{\phi_{J},A}}\;[\text{exp}\;(\;-\frac{r_{\text{M}}^{\alpha }\Gamma_{\text{M}}}{P_{t,\nu }^{\text{UL}}}\;(I_{\phi_{\text{M}},A_{2}}^{\text{UL}}\;+\;I_{\phi_{\text{S}},A_{1}}^{\text{DL}}\;+\;I_{\phi_{J},A})\;)\;]\\ \overset{\left(3\right)}{=}{\text{E}}_{r_{\text{M}}}\;[L_{I_{\phi_{\text{M}},A_{2}}^{\text{UL}}}\;(s)\times L_{I_{\phi_{\text{S}},A_{1}}^{\text{DL}}}(s)\times L_{I_{\phi_{J},A}}(s)]{|}_{s=\frac{r_{\text{M}}^{\alpha }\Gamma_{\text{M}}}{P_{t,\nu }^{\text{UL}}}}. \end{array}$$ (4)

Here, Step (1) follows from the coverage probability definition [17, 40]. Step (2) follows from Step (1) by using the void property of IHPPPs [40]. Similarly, Step (3) is obtained by replacing $\frac{r_{\text{M}}^{\alpha }\Gamma_{\text{M}}}{P_{t,\nu }^{\text{UL}}}$ by *s*, where $s=\frac{r_{\text{M}}^{\alpha }\Gamma_{\text{M}}}{P_{t,\nu }^{\text{UL}}}$. In addition, Stage (4) is obtained by the use of the exponential property of additions in products i.e., exp(*a* + *b*) = exp(*a*) × exp(*b*).

The Laplace transform (LT) of interference in UL from MBSs in *A*_2_, i.e., $L_{I_{\phi_{\text{M}},A_{2}}^{\text{UL}}}$, is obtained as:

$$\begin{array}{cc} & L_{I_{\phi_{\text{M}},A_{2}}^{\text{UL}}}(s)\overset{\left(a\right)}{=}{\text{E}}_{I_{\phi_{\text{M}},A_{2}}^{\text{UL}}}[\text{exp}(-I_{\phi_{\text{M}},A_{2}}^{\text{UL}}s)]{|}_{s=\frac{r_{\text{M}}^{\alpha }\Gamma_{\text{M}}}{P_{t,\nu }^{\text{UL}}}}\\ & \overset{\left(b\right)}{=}{\text{E}}_{I_{\phi_{\text{M}},A_{2}}^{\text{UL}},{\left|h_{l}\right|}^{2}}[\text{exp}(-s\sum_{l\in \phi_{\text{M}}}^{}P_{t,\nu }^{\text{UL}}{\left|h_{l}\right|}^{2}r_{l}^{-\alpha })]\\ & \overset{\left(c\right)}{=}{\text{E}}_{I_{\phi_{\text{M}},A_{2}}^{\text{UL}},{\left|h_{l}\right|}^{2}}[\prod_{l\in \phi_{\text{M}}}^{}\text{exp}(-|h_{l}{|}^{2}\Gamma_{\text{M}}r_{\text{M}}^{\alpha }r_{l}^{-\alpha })] \end{array}$$

$$\begin{array}{cc} & \overset{\left(d\right)}{=}{\text{E}}_{I_{\phi_{\text{M}},A_{2}}^{\text{UL}}}[\prod_{l\in \phi_{\text{M}}}{\text{E}}_{|h_{l}{|}^{2}}\text{exp}(-|h_{l}{|}^{2}\Gamma_{\text{M}}r_{\text{M}}^{\alpha }r_{l}^{-\alpha })]\\ & \overset{\left(e\right)}{=}{\text{E}}_{I_{\phi_{\text{M}},A_{2}}^{\text{UL}}}[\prod_{l\in \phi_{\text{M}}}\frac{1}{1+\Gamma_{\text{M}}{\left(\frac{r_{l}}{r_{\text{M}}}\right)}^{-\alpha }}]\\ & \overset{\left(f\right)}{=}\text{exp}(-2\pi \zeta_{\text{M}}\int_{\Delta s1}^{\Delta s_{2}}\frac{r_{l}dr_{l}}{1+{\left(\frac{r_{l}}{\Gamma_{\text{M}}^{1/\alpha }r_{\text{M}}}\right)}^{\alpha }})\\ & \overset{\left(g\right)}{=}\text{exp}(\;-\pi \zeta_{\text{M}}\Gamma_{\text{M}}^{2/\alpha }r_{\text{M}}^{2}\int_{{\left(\frac{\Delta s_{1}}{\Gamma_{\text{M}}^{1/\alpha }r_{\text{M}}}\right)}^{2}}^{{\left(\frac{\Delta s_{2}}{\Gamma_{\text{M}}^{1/\alpha }r_{\text{M}}}\right)}^{2}}\frac{du}{1+{\left(u\right)}^{\alpha /2}})\\ & \overset{\left(h\right)}{=}\text{exp}(\frac{\zeta_{\text{M}}\pi \Gamma_{\text{M}}\Delta s_{2}^{\left(2-\alpha \right)}r_{\text{M}}^{\alpha }}{\alpha /2-1}{}_{2}F_{1}(1,1-\frac{2}{\alpha },2-\frac{2}{\alpha },-\Gamma_{\text{M}}{\left(\frac{r_{\text{M}}}{\Delta s_{2}}\right)}^{\alpha })-\\ & \frac{\zeta_{\text{M}}\pi \Gamma_{\text{M}}\Delta s_{1}^{\left(2-\alpha \right)}r_{\text{M}}^{\alpha }}{\alpha /2-1}{}_{2}F_{1}(1,1-\frac{2}{\alpha },2-\frac{2}{\alpha },-\Gamma_{\text{M}}{\left(\frac{r_{\text{M}}}{\Delta s_{1}}\right)}^{\alpha })). \end{array}$$ (5)

Here, Step (*a*) follows the definition of LT [40], Step (*b*) is achieved by substituting $I_{\phi_{\text{M}},A_{2}}^{\text{UL}}=\sum_{l\in \phi_{\text{M}}}\;P_{t,l}^{\text{UL}}\left|h_{l}\right|r_{l}^{-\alpha }\;$, into Step (*a*), Step (*c*) is achieved by replacing *s*, s.t., $s=\frac{r_{\text{M}}^{\alpha }\Gamma_{\text{M}}}{P_{t,\nu }^{\text{UL}}}$, into Step (*b*), Step (*e*) is followed by evaluating the LT of Step (*d*) with respect to *h*_*j*_, Step (*f*), is followed by considering probability generating functional (PGFL) of IHPPP [47], Step (*g*) is achieved by replacing $u={\left(\frac{r_{j}}{{\left(\Gamma_{\text{M}}\right)}^{1/\alpha }r_{\text{M}}}\right)}^{2}$ into Step (*f*), and Step (*h*) is achieved from Gauss-hypergeometric approximation of Step (*g*) [47].

Similarly, the LT of the total UL interference received from the MBSs in *A*_1_, i.e., $L_{I_{\phi_{\text{M}},A_{1}}^{\text{UL}}}(s)$, is obtained as:

$$\begin{array}{cc} & L_{I_{\phi_{\text{M}},A_{2}}^{\text{UL}}}(s)\\ & =\text{exp}(\frac{\zeta_{\text{M}}\pi \Gamma_{\text{M}}\Delta s_{2}^{\left(2-\alpha \right)}r_{\text{M}}^{\alpha }}{\alpha /2-1}{}_{2}F_{1}(1,1-\frac{2}{\alpha },2-\frac{2}{\alpha },-\Gamma_{\text{M}}{\left(\frac{r_{\text{M}}}{\Delta s_{2}}\right)}^{\alpha })\\ & -\frac{\zeta_{\text{M}}\pi \Gamma_{\text{M}}\Delta s_{1}^{\left(2-\alpha \right)}r_{\text{M}}^{\alpha }}{\alpha /2-1}{}_{2}F_{1}(1,1-\frac{2}{\alpha },2-\frac{2}{\alpha },-\Gamma_{\text{M}}{\left(\frac{r_{\text{M}}}{\Delta s_{1}}\right)}^{\alpha })). \end{array}$$ (6)

In addition, the LT of the DL interference from SBSs in *A*_1_, i.e., $L_{I_{\phi_{\text{S}},A_{1}}^{\text{DL}}}$, can be written in a similar way as far (5), and is given as:

$$\begin{array}{cc} & L_{I_{\phi_{\text{S}},A_{1}}^{\text{DL}}}=\\ & \text{exp}(\frac{\zeta_{\text{S}}^{{}^{\prime }}\pi \eta_{3}\Gamma_{\text{M}}x_{2}^{\left(2-\alpha \right)}r_{\text{M}}^{\alpha }}{\alpha /2-1}{}_{2}F_{1}(1,1-\frac{2}{\alpha },2-\frac{2}{\alpha },-\eta_{3}\Gamma_{\text{M}}{\left(\frac{r_{\text{M}}}{x_{2}}\right)}^{\alpha })-\\ & \frac{\zeta_{\text{S}}^{{}^{\prime }}\pi \eta_{3}\Gamma_{\text{M}}x_{1}^{\left(2-\alpha \right)}r_{\text{M}}^{\alpha }}{\alpha /2-1}{}_{2}F_{1}(1,1-\frac{2}{\alpha },2-\frac{2}{\alpha },-\eta_{3}\Gamma_{\text{M}}{\left(\frac{r_{\text{M}}}{x_{1}}\right)}^{\alpha })). \end{array}$$ (7)

$$\begin{array}{cc} & L_{I_{\phi_{J},A}}(s)=\\ & \text{exp}(\frac{\zeta_{j}\pi \eta_{2}\Gamma_{\text{M}}z_{2}^{\left(2-\alpha \right)}r_{\text{M}}^{\alpha }}{\alpha /2-1}{}_{2}F_{1}(1,1-\frac{2}{\alpha },2-\frac{2}{\alpha },-\eta_{2}\Gamma_{\text{M}}{\left(\frac{r_{\text{M}}}{z_{2}}\right)}^{\alpha })-\\ & \frac{\zeta_{j}\pi \eta_{2}\Gamma_{\text{M}}z_{1}^{\left(2-\alpha \right)}r_{\text{M}}^{\alpha }}{\alpha /2-1}{}_{2}F_{1}(1,1-\frac{2}{\alpha },2-\frac{2}{\alpha },-\eta_{2}\Gamma_{\text{M}}{\left(\frac{r_{\text{M}}}{z_{1}}\right)}^{\alpha })). \end{array}$$ (8)

*η*_2_ is the ratio of $P_{t,\text{S}}^{\text{DL}}$ and $P_{t,\nu }^{\text{UL}}$ where $P_{t,\text{S}}^{\text{DL}}$ is the DL transmit power of SBSs.

The UL coverage probability, $P_{A_{2}}^{\text{UL},*}(\Gamma_{\text{M}})$, in the presence of ICI, IJI, and RFA employment while considering *ν* in *A*_2_ can be written as [17]

$$\begin{array}{cc} & P_{A_{2}}^{\text{UL},*}(\Gamma_{\text{M}})=\int_{\Delta s_{1}}^{d_{2}}L_{I_{\phi_{\text{M}},A_{2}}^{\text{UL}}}(s)\times L_{I_{\phi_{\text{S}},A_{1}}^{\text{DL}}}(s)\times L_{I_{\phi_{J},A}}(s)\\ & f_{r_{\text{M},\nu }|\nu_{A_{2}}}(r_{\text{M},\nu })dr_{\text{M},\nu }. \end{array}$$ (9)

$$\begin{array}{cc} & \;P_{A_{2}}^{\text{UL},*}\left(\Gamma_{\text{M}}\right)={\frac{2\pi \zeta_{\text{M}}}{\text{exp}(-\zeta_{\text{M}}\pi d_{1}^{2})}}_{\Delta s_{1}}^{\Delta s_{2}}\text{exp}(\frac{\pi \Gamma_{\text{M}}r_{\text{M}}^{\alpha }}{\alpha /2-1}[\zeta_{\text{M}}\Delta s_{2}^{\left(2-\alpha \right)}\;J\;\left(\;\alpha ,\;-\Gamma_{\text{M}}\;{\left(\frac{r_{\text{M}}}{\Delta s_{2}}\right)}^{\;\alpha }\right)\;-\;\zeta_{\text{M}}{\Delta s_{1}}^{\left(2-\alpha \right)}J\;\left(\;\alpha ,\;-\Gamma_{\text{M}}\;{\left(\frac{r_{\text{M}}}{\Delta s_{1}}\right)}^{\;\alpha }\right)\;.\\ & \;+\zeta_{\text{S}}^{{}^{\prime }}\eta_{3}\Delta s_{1}^{\left(2-\alpha \right)}J\left(\alpha ,-\Gamma_{\text{M}}\eta_{3}{\left(\frac{r_{\text{M}}}{\Delta s_{1}}\right)}^{\alpha }\right)-\zeta_{\text{S}}^{{}^{\prime }}\eta_{3}y^{\left(2-\alpha \right)}J\left(\alpha ,-\Gamma_{\text{M}}\eta_{3}{\left(\frac{r_{\text{M}}}{y}\right)}^{\alpha }\right)+\zeta_{j}\eta_{2}\Delta s_{2}^{\left(2-\alpha \right)}J\left(\alpha ,-\Gamma_{\text{M}}\eta_{2}{\left(\frac{r_{\text{M}}}{\Delta s_{2}}\right)}^{\alpha }\right)\\ & \;-\zeta_{j}\eta_{2}y^{\left(2-\alpha \right)}J\left(\alpha ,-\Gamma_{\text{M}}\eta_{2}{\left(\frac{r_{\text{M}}}{y}\right)}^{\alpha }\right)]-\zeta_{\text{M}}\pi r_{\text{M}}^{2})r_{\text{M}}dr_{\text{M}}. \end{array}$$ (10)

$$\begin{array}{cc} & \;P_{A_{2}}^{\text{UL}}(\Gamma_{\text{M}})={\frac{2\pi \zeta_{\text{M}}}{\text{exp}(-\zeta_{\text{M}}\pi d_{1}^{2})}}_{\Delta s_{1}}^{\Delta s_{2}}\text{exp}(\frac{\pi \Gamma_{\text{M}}r_{\text{M}}^{\alpha }}{\alpha /2-1}[\zeta_{\text{M}}\Delta s_{2}^{\left(2-\alpha \right)}\;J\;(\;\alpha ,\;-\Gamma_{\text{M}}\;{\left(\frac{r_{\text{M}}}{\Delta s_{2}}\right)}^{\;\alpha })\;-\zeta_{\text{M}}{\Delta s_{1}}^{\left(2-\alpha \right)}J\;(\;\alpha ,\;-\Gamma_{\text{M}}\;{\left(\frac{r_{\text{M}}}{\Delta s_{1}}\right)}^{\;\alpha })\\ & \;+\zeta_{j}\eta_{2}\Delta s_{2}^{\left(2-\alpha \right)}J(\alpha ,-\Gamma_{\text{M}}\eta_{2}{\left(\frac{r_{\text{M}}}{\Delta s_{2}}\right)}^{\alpha })-\zeta_{j}\eta_{2}y^{\left(2-\alpha \right)}J(\alpha ,-\Gamma_{\text{M}}\eta_{2}{\left(\frac{r_{\text{M}}}{y}\right)}^{\alpha })]-\zeta_{\text{M}}\pi r_{\text{M}}^{2})r_{\text{M}}dr_{\text{M}}. \end{array}$$ (11)

By substituting (6), (7), and (8) into (9), $P_{A_{2}}^{\text{UL},*}(\Gamma_{\text{M}})$ is expressed as (10).

### 3.2 Uplink coverage probability for non-uniform HetNets (NUHs’)

Non-uniform heterogeneous network deployment is established where SBS in *A*_1_ is muted and user in that vicinity is in coverage with MBS. The UL coverage probability, $P_{A_{2}}^{\text{UL}}(\Gamma_{\text{M}})$, while assuming IJs, RFA, and *ν* in *A*_2_ can be written as

$$\begin{array}{cc} & P_{A_{2}}^{\text{UL}}(\Gamma_{\text{M}})=\;\int_{\Delta s_{1}}^{d_{2}}\;L_{I_{\phi_{\text{M}},A_{2}}^{\text{UL}}}\;(s)\times L_{I_{\phi_{J},A}}(s)f_{r_{\text{M},\nu }|\nu_{A_{2}}}(r_{\text{M},\nu })dr_{\text{M},\nu }. \end{array}$$ (12)

By substituting (6) and (8) into (12), $P_{A_{1}}^{\text{UL}}(\Gamma_{\text{M}})$ is expressed as (11). In (10) and (11), J(·) indicates the Gauss-hypergeometric function.

## 4 Results and discussion

This section describes results for the user’s UL coverage probability while taking into consideration: (i) UL coverage probability of UH and (ii) UL coverage probability of NUH. MATLAB 2015a has been used in drawing our results. MBS, SBS, jammers and users are dispersed in *A* = *π*(500m)^2^, s.t., *A* = *A*_1_U*A*_2_. Transmitted power by MBS, SBS, jammers, and users are supposed to be 40 dBm, 30 dBm, 20 dBm, and 20 dBm, respectively. Various network parameters such as *ζ*_M_, *ζ*_S_, *ζ*_*j*_, Γ_M_, *P*_*t*,*J*_ and $P_{t,u}^{\text{UL}}$ are assumed for analyzing UL coverage when the user is located in *A*_2_.

**Fig 3:**
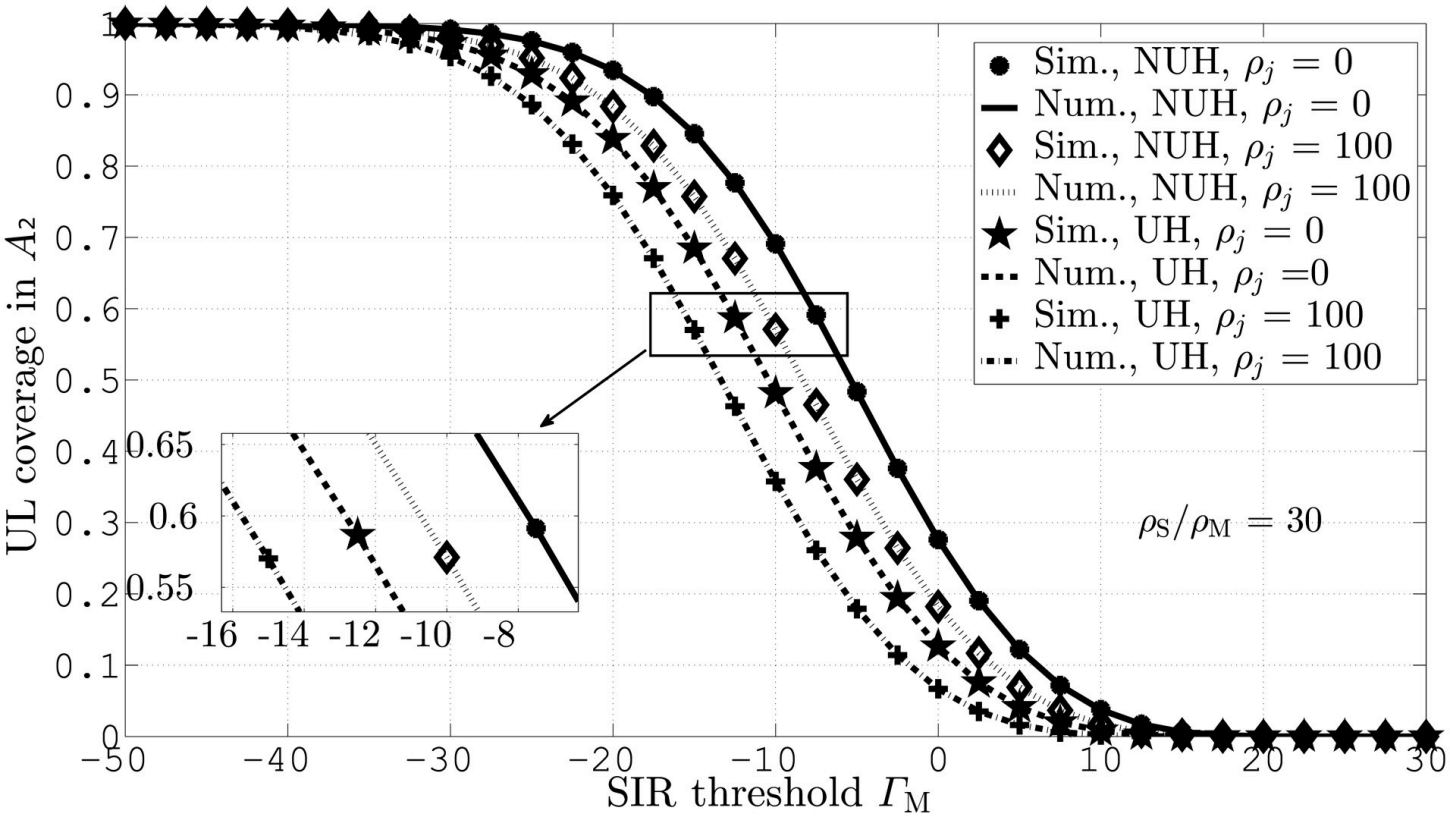
UL coverage probabilities against Γ_M_ and *ζ*_*j*_ in *A*_2_.

Fig 3 compares UL coverage probability for different values of Γ_M_ in *A*_2_. This figure assumes *ζ*_*j*_ = 0 and 100, for both UH and NUH network scenarios. This figure indicates that the simulation results will coincide with the numerical results both for UH and NUH. The plots in the figure further demonstrate that NUHs with *ζ*_*j*_ = 0 lead to the highest coverage gain as compared with the rest of the scenarios. This is due to improved interference mitigation by NUHs as a result of lower SBS deployment.

**Fig 4:**
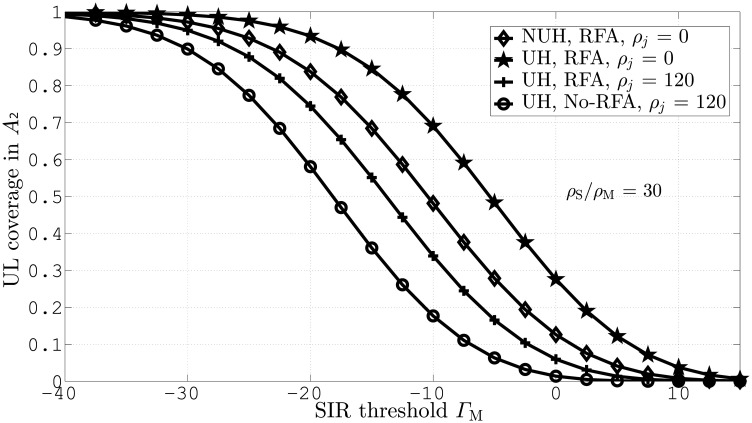
UL coverage probabilities for UH and NUH in *A*_2_.

In Fig 4, we demonstrate UL coverage probability against different values of Γ_M_ for both UH and NUH in *A*_2_. This figure is obtained for *ζ*_*j*_ = 0 and 100 and *ζ*_S_/*ζ*_M_ = 30. This result demonstrates that NUH with RFA outperforms the other scenarios due to significant interference mitigation. At Γ_M_ = −10*dB*, the proposed NUH with RFA and *ζ*_*j*_ = 0 leads to 20% UL coverage gain.

**Fig 5:**
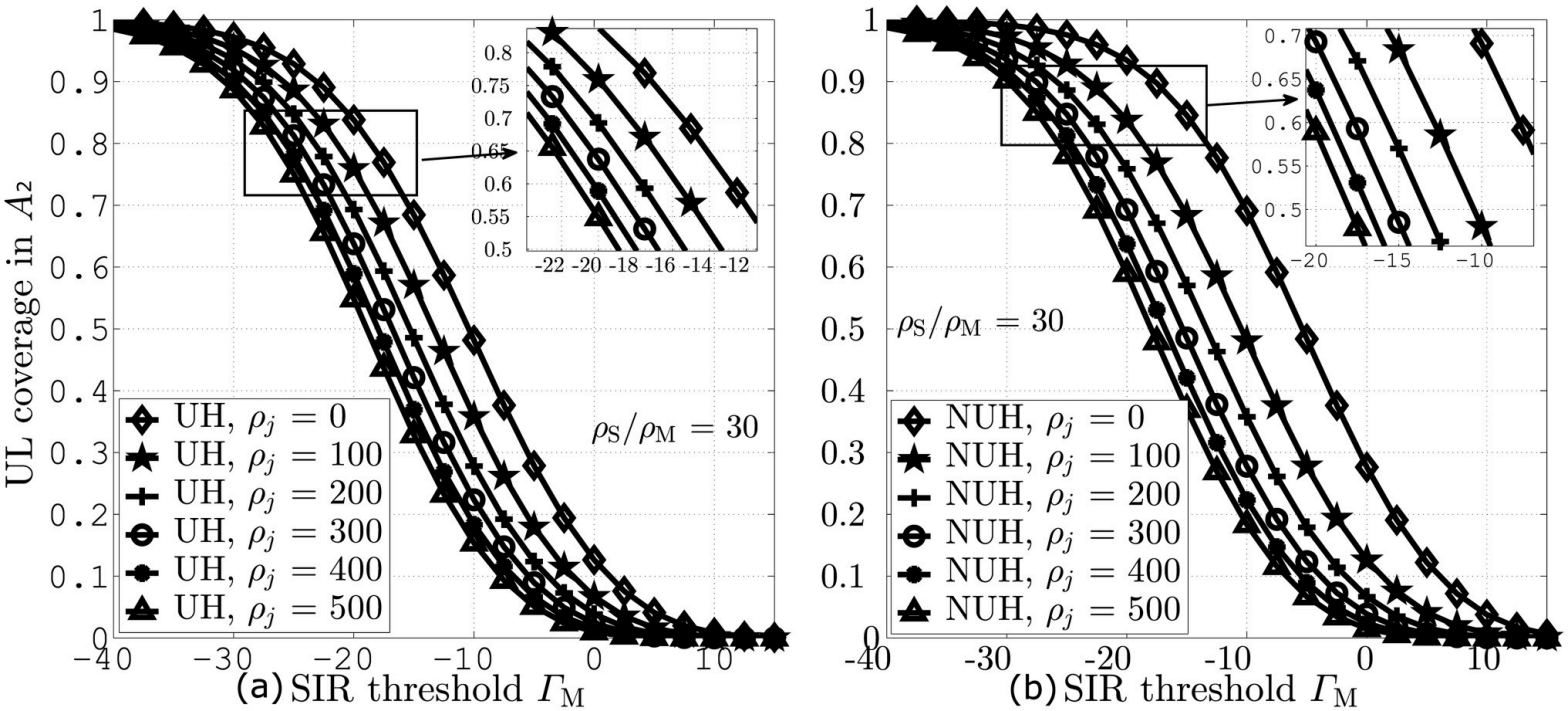
UL coverage probabilities against Γ_M_ and *ζ*_*j*_. A: UH B: NUH.

In Fig 5A and 5B, we compare UL coverage probability against different values of Γ_M_ for UH and NUH, respectively. The plots in both the figures are obtained for *ζ*_*j*_ = 0, 100, 200, 300, 400, 500. Moreover, the results indicate that a sufficient number of IJs in the network are needed to significantly degrade UL coverage because of the wideband nature and low transmission power of IJs. Furthermore, increasing the value *ζ*_*j*_ leads to lower UL coverage in both UH and NUH due to higher interference. The results indicate significant coverage performance improvements by RFA and NUH due to effective interference mitigation.

**Fig 6:**
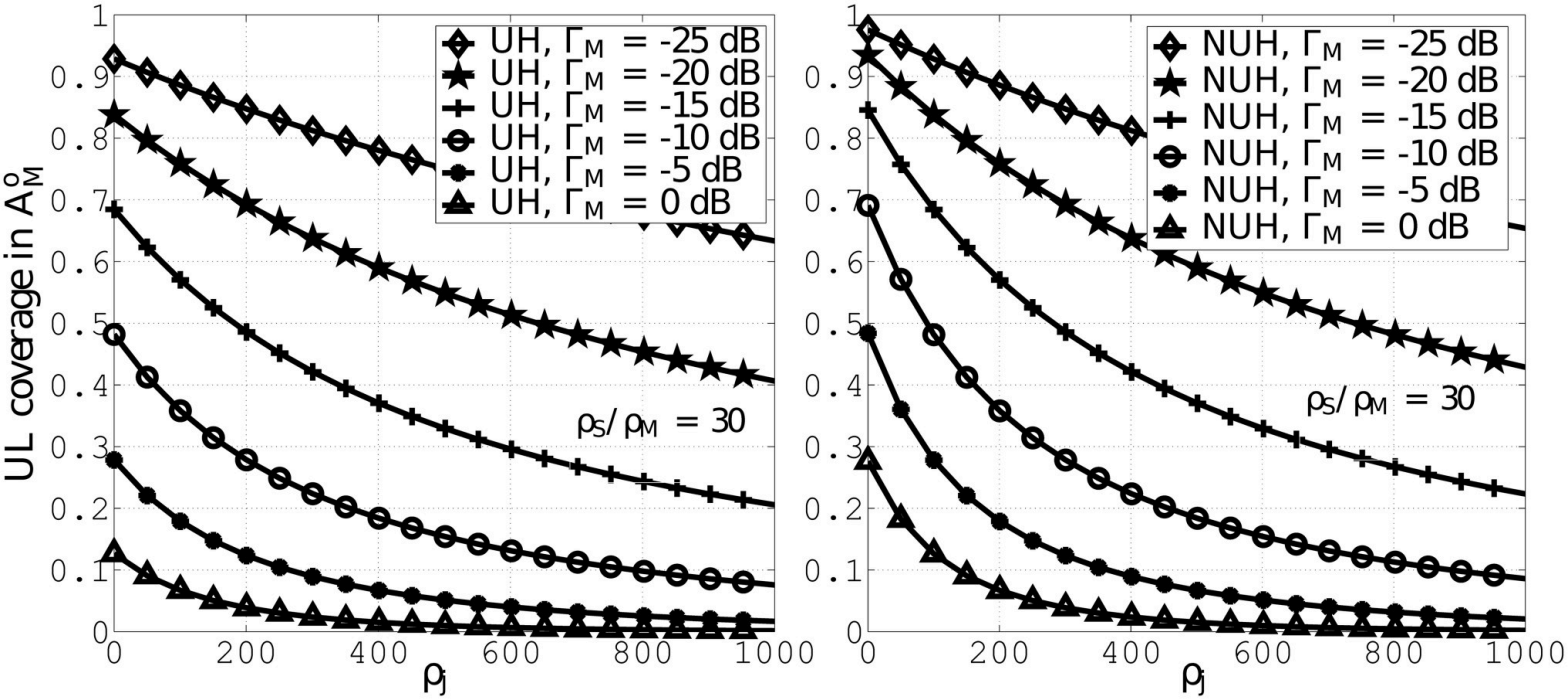
UL coverage probabilities against *ζ*_*j*_. A: UH B: NUH.

In Fig 6A and 6B, we evaluate UL coverage probability for different values of *ζ*_*j*_, while considering RFA, UH, and NUH. The plots are obtained for Γ_M_ = 0 dB, −5 dB, −10 dB, −15 dB, −20 dB, −25 dB and *ζ*_S_/*ζ*_M_ = 30. The plots indicate that higher values of Γ_M_ lead to lower coverage due to lower user association. Furthermore, the plots in both figures indicate that NUH gives rise to higher coverage in contrast to UH. By employing RFA, the network performance improves in both cases but due to less interference the coverage in NUH is better than UH.

**Fig 7:**
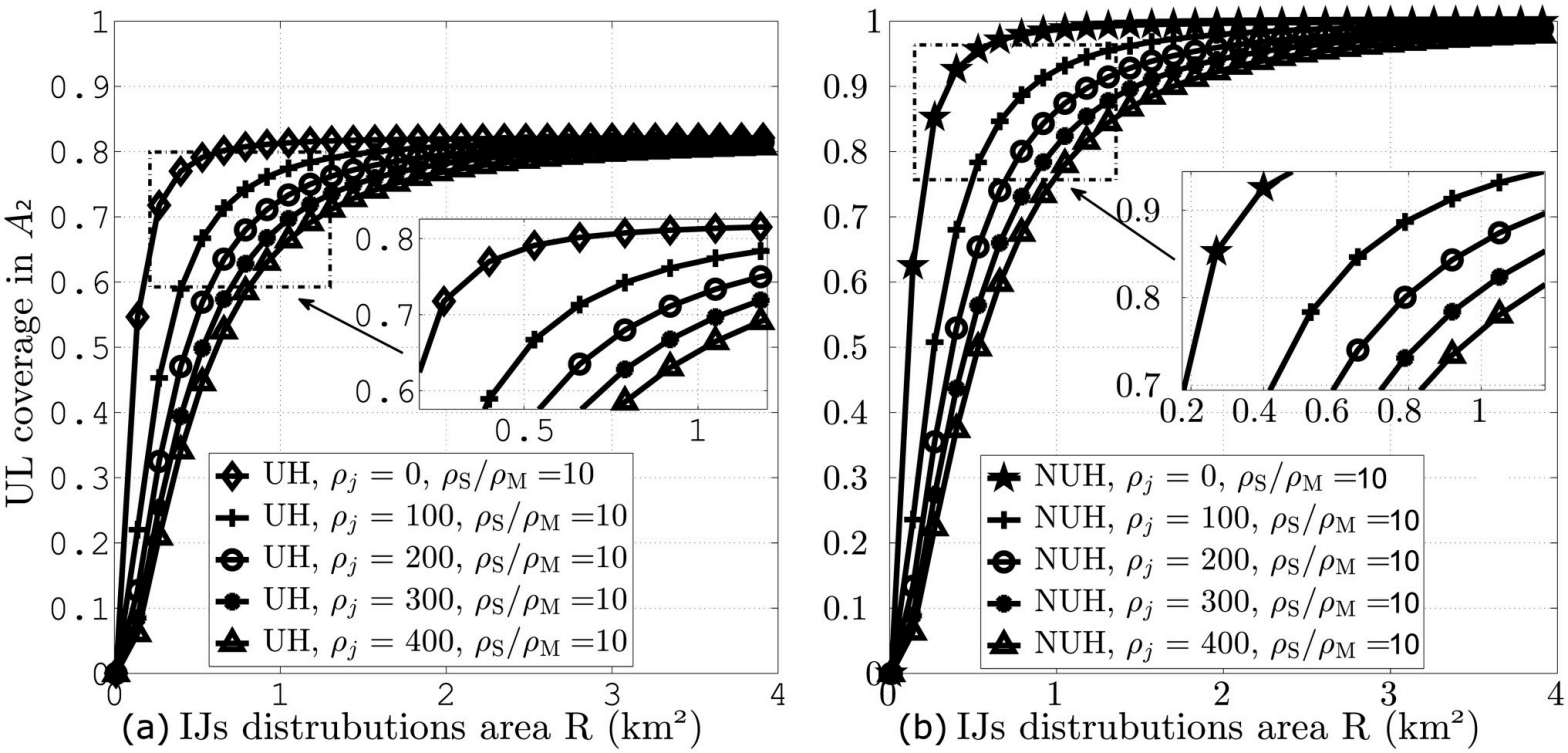
UL coverage probabilities against IJs distribution area for different values of *ζ*_*j*_ and *ζ*_S_/*ζ*_M_ = 10. A: UH B: NUH.

Similarly, Fig 7A and 7B demonstrate UL coverage performance against IJs distribution area for different values of *ζ*_*j*_ and *ζ*_S_/*ζ*_M_ = 10.

Both of these figures indicate that increasing IJs distribution area leads to improved coverage as the IJs become less effective. The figures further depict that at an area of 1 km^2^, NUH with RFA leads to 19% UL coverage improvement due to significant interference reduction.

**Fig 8:**
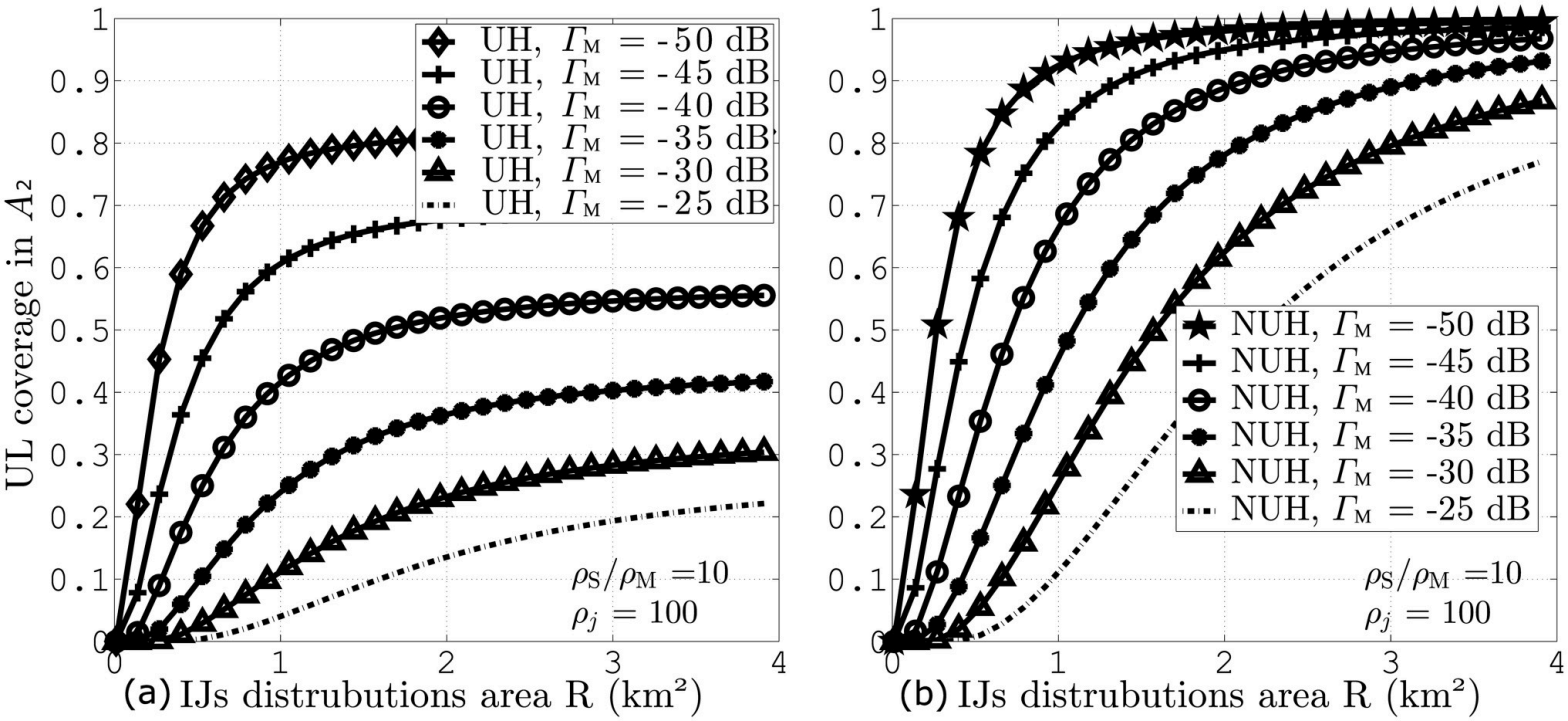
UL coverage probabilities against IJs distribution area for different values of Γ_M_ and *ζ*_*j*_ = 100. A: UH B: NUH.

Finally, Fig 8A and 8B show UL coverage probability against IJs distribution area for different values of Γ_M_. These figures consider *ζ*_*j*_ = 100 and *ζ*_S_/*ζ*_M_ = 10. The results indicate that increase in the values of Γ_M_ gives rise to lower coverage due to lower user connection. The figures also indicate that NUH with RFA and Γ_M_ = -50 dB give rise to the highest coverage gain in contrast to the rest of the scenarios.

## 5 Conclusion

This work aims to reduce ICI and IJI by employing SBS muting and RFA in HetNets. Various network parameters such as jammer’s density, jammers transmit power and their distribution area, SIR threshold are investigated against user coverage. The results are obtained for both UHs and NUHs in addition to and without RFA. The results depict that NUHs employing RFA outperform other scenarios in terms of UL coverage. Moreover, the investigation indicates 20% UL coverage improvement at Γ_M_ = −10 dB while using RFA and NUHs as compared with RFA and UHs. This work can be extended to evaluate drone-based jammers in HetNets.
